# New twists in the evolution of retinal direction selectivity

**DOI:** 10.1371/journal.pbio.3002538

**Published:** 2024-02-29

**Authors:** Takeshi Yoshimatsu, Tom Baden

**Affiliations:** 1 Department of the Ophthalmology and Visual Sciences, Washington University in St. Louis School of Medicine, St. Louis, Missouri, United States of America; 2 Center for Sensory Neuroscience and Computation, Sussex Neuroscience, School of Life Sciences, University of Sussex, Sussex, United Kingdom

## Abstract

In mammals, starburst amacrine cells are centrally involved in motion vision. This primer explores the implications of a PLOS Biology study reporting that zebrafish have amacrine cells, too, and revealing that these cells coexist with a second pair of starburst-like neurons, but neither appears to be strongly motion selective.

## Teaser

In mammals, starburst amacrine cells are centrally involved in motion vision and a new study in *PLOS Biology*, by Yan and colleagues finds that zebrafish have them, too. They coexist with a second pair of starburst-like neurons, but neither appears to be strongly motion selective.

The evolutionary origins of the vertebrate retina date back more than 500 million years, to a time when the first would-be vertebrates abandoned their previously stationary lifestyles and took to the open water [1,2]. Our early ancestors would have had to rapidly acquire the ability to stabilize in the water, to navigate, and to detect predators and prey—all requirements that in one way or another depend on the detection of visual motion. Consequently, it seems reasonable to speculate that motion circuits are an ancestral trait of the vertebrate eye.

Indeed, the eyes of all sighted extant vertebrates share a common retinal circuit blueprint: Five classes of neurons, arranged in 3 nuclear layers flanking 2 synaptic layers. This much has been clear for well over a century [3]. However, except for mammals, what microcircuits sit inside each retinal layer of different species remains largely unknown, leaving us all but blind to much of the evolutionary past of what might be our most valued sense. Are some or even most of the neurons and circuits that sit within the various retina layers of a mouse or human the same that sit in the eyes of a bird, a frog, or a fish? And if so, do such “orthologous circuits” perform orthologous tasks?

A new study by Yan and colleagues [4] sheds critical new light into this surprisingly underexplored world: Starburst amacrine cells, integral parts of mammalian retinal circuits involved in the detection of directional motion [5], are present in zebrafish!

**Fig 1:**
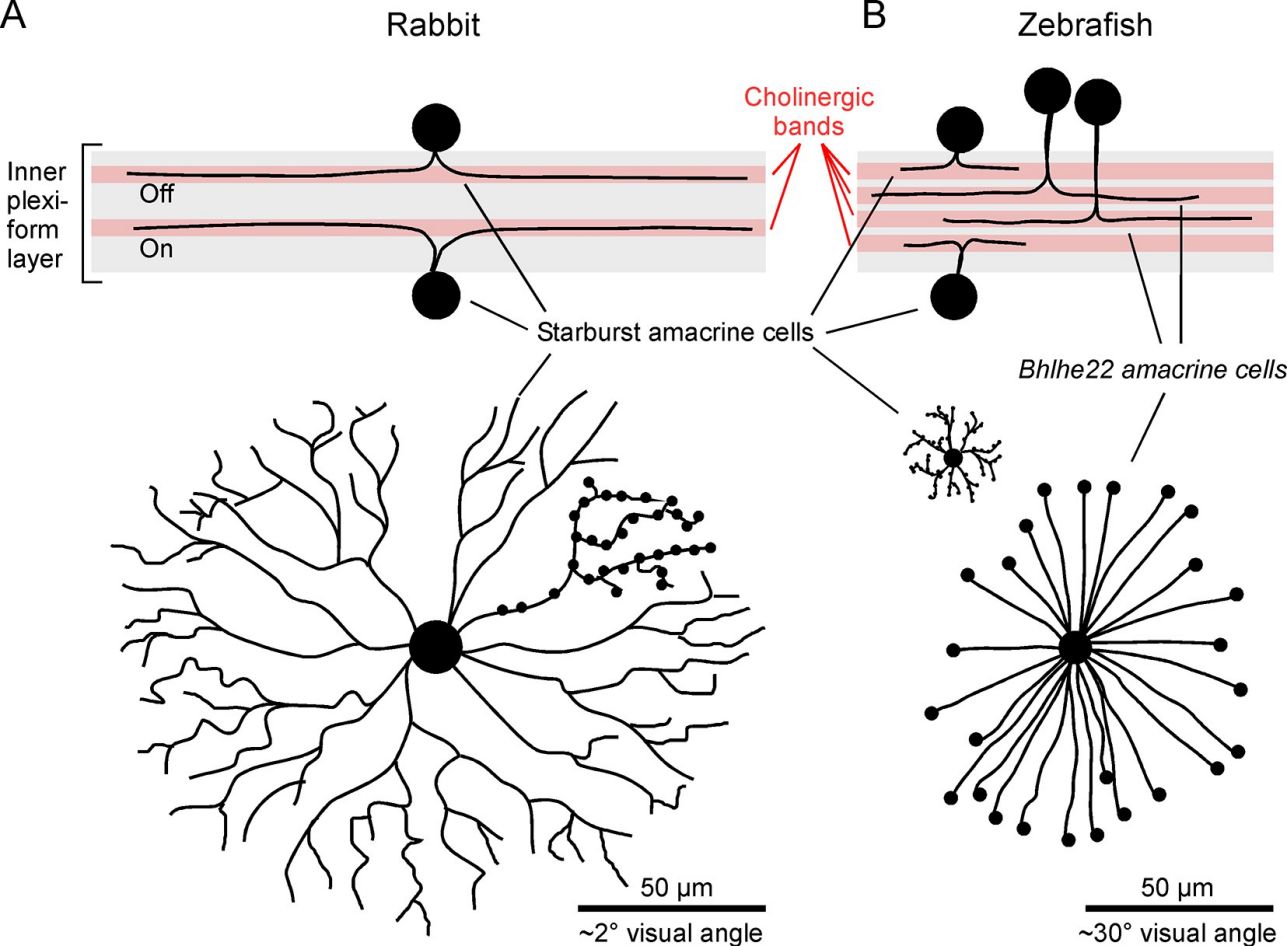
Cholinergic amacrine cells of mice and zebrafish. (**A**) Cross-sectional view of rabbit inner retina, with cholinergic bands and anatomy of starburst cells indicated (top) and en-face schematic of a single rabbit starburst cell (redrawn based on [5]). (**B**) As (A), but for larval zebrafish (redrawn based on [4]) showing their much more compact starburst cells alongside the newly discovered bhlhe22 neurons: a pair of notably larger, radially organized cholinergic amacrine cells that appear to be absent in mammals. Scale bars given in both absolute size and in visual angle: in the <0.3 mm diameter eye of larval zebrafish, already small dendrites cover large areas in visual space. “Blobs” on dendrites indicate putative synaptic boutons.

Starburst cells, as we know them from mammals, (Fig 1A) [6] are equally enigmatic and weird. Lacking an axon, they are flat like a pancake with dendrites radiating outwards from a central soma like an exploding star (hence the name). They are also dual-transmitter neurons, releasing both GABA and acetylcholine. While the former is critical for motion circuits, starburst cells are traditionally defined based on their cholinergic profile: Many amacrine cells release GABA, but in mammals, only starburst cells release acetylcholine. Most if not all mammalian retinas display 2 “ChAT-bands” (Fig 1A, top): highly specific strata of the inner retina that are immunoreactive for choline acetyltransferase (ChAT). These ChAT-bands correspond 1:1 to the dendrites of 2 types of mammalian starburst cells (“Off” and “On”), and from a circuit perspective, the 2 bands represent *the* central locus for direction selective circuits [7], starting with the presynaptic inputs from retinal bipolar cells, and ending with the dendritic processes of directionally selective retinal ganglion cells that carry the extracted motion signals to the brain. Correspondingly, ChAT bands and their associated circuits have been a central focus of efforts aiming to understand how eyes process motion, and today they represent the perhaps best understood complex circuit of the vertebrate brain.

So, what of zebrafish? First, the presence of bona fide starburst cells in their eyes [4] (Fig 1B) strongly indicates that these cells are old: at least 400 million years, when the zebrafish’s ray finned ancestors split from early lobe finned fish that would eventually give rise to all tetrapods which include mammals alongside all amphibians, birds, and reptiles [1]. Correspondingly, this also means that probably all these other tetrapods have starburst cells, and if not, it would mean that these cells were lost rather than that they never existed. The presence of starburst cells in the eyes of most if not all vertebrates was long suspected—after all, vertebrate retinas consistently have ChAT [8,9]. However, it had remained unclear if these cholinergic bands correspond to starburst cells. Moreover, while most mammals have 2 bands, non-mammals often have more: Three or even 4 ChAT bands are common from fish to birds. Do we therefore expect up to 4 types of starburst cells in their eyes?

Here comes the next major insight from the new work. No, there are still just the same familiar 2 types of starburst cells in the eyes of zebrafish. However, a second pair of cholinergic neurons, located in between the starburst cells, corresponds to a new type of amacrine cell, dubbed “bhlhe22” (Fig 1B, top). Not only are these extra cells dual GABA/acetylcholine neurons, but they also resemble key aspects of a “starburst anatomy” (Fig 1B, bottom). If anything, they look even more like an exploding star compared to the original. A soma in the middle, and some 20 to 30 unbranched and perfectly straight dendrites radiating outwards, each with a little blob—presumably a synaptic bouton—on the end. Mammalian starburst dendrites are curvier and more branched (Fig 1A, bottom).

In mammals, the starburst cells’ radial organization is fundamental to their function. Each dendrite is direction selective along its own orientation, achieved by a cornucopia of mechanisms that exploit kinetic and electronic differences along the dendrite’s input–output organization [5,7]. A consequence of this dendrite-oriented selectivity is that postsynaptic circuits “simply” grab only the dendrites that go in the “right” direction to build a direction-selective circuit for any axis [10]. Looking at the zebrafish starburst orthologs (Fig 1B, bottom left), it remains unclear how the same computation could work. In the tiny eye of larval zebrafish, starburst cells are compact, and while their radial organization is not entirely lost, it is perhaps also not their most obvious feature. This is compounded with the fact that despite their mini-dendrites, larval zebrafish starburst cells end up covering some 15° of visual space, more than 3 times the angle subtended by the starburst cells in the eyes of most mammals [11]. Potentially in line, functional measurements from the dendrites of fish starburst cells hint at some direction selectivity, however compared to the strong selectivity found in the mammals [5], the fish version seems underwhelming. The dendritic signals from bhlhe22 cells also showed directional responses, but, again, selectivity was weak. While it remains possible that these results can be explained by technical limitations, it could also simply mean exactly what it says on the tin: that retinal direction selectivity, at least in zebrafish, does not primarily come from starburst cells, or even from cholinergic circuits. By extension, this would also imply that the radial organization of these circuits originally evolved in response to selection pressures other than those associated with the need to achieve motion vision.

The fact that starburst cells are part-cholinergic is generally confusing. Unlike GABAergic signaling, which is point-to-point, cholinergic signals are thought to be more diffuse, which seems ill-suited to convey information about the localized direction of motion (but see [12]): Signals from differently oriented dendrites should mix and thus lose selectivity. Why then hang on to the acetylcholine signal at all? Here, perhaps the cholinergic neurons of zebrafish will eventually lead us towards a satisfactory answer. Perhaps zebrafish starburst and/or bhlhe22 cells retain whatever the ancestral cholinergic functions might have been. Moreover, the putative absence of strong directionally selective responses in zebrafish cholinergic circuits also hints that in their eyes, distinct and potentially more ancestral mechanisms exist for this purpose.

Taking a step back, we are currently in a time of great opportunity in the field of neural circuit evolution, specifically using the ancient circuits of the vertebrate retina as a playground. Only a few months ago learned that within mammals, and perhaps beyond, most retinal neurons can be molecularly linked across all extant species [13]. Already the jawless lampreys, representatives of the evolutionarily most distant extant vertebrates, feature transcriptomic signatures of a great many usual-suspect neurons—notably including not 2 but 4 transcriptomic signatures of cholinergic amacrine cells [9]! Another recent preprint [14] reported the zebrafish ortholog of the mammalian rod bipolar cell, plus their associated circuits, which together underpin our own sense of night vision. Mirroring the findings from the current new study on zebrafish cholinergic amacrine cells [4], there are again 2 types. The “mammal one,” and another version that is absent in mammals. Understanding what these types of “extra” neurons contribute to the eyes of fish, or why they appear to be absent in the eyes of mammals, will be fascinating to explore in the future.
